# The challenge of composite graft: the use of fluorescent light energy to improve engraftment

**DOI:** 10.1080/23320885.2024.2396946

**Published:** 2024-08-27

**Authors:** Antonio Amabile, Stefano L’Erario, Carlotta Scarpa, Vincenzo Vindigni, Franco Bassetto, Francesco Marena

**Affiliations:** Department of Neurosciences, University Hospital of Padua, Padova, Italy

**Keywords:** Composite graft, fluorescent light energy, facial aesthetic subunits, alar reconstruction, FLET

## Abstract

Composite graft is a useful tool for the reconstruction of specific facial aesthetic subunits with a single surgical stage. This technique, when successful, gives optimal results in the reconstruction of small defects of nose, ear, eyelid and lips. The aim of this work is to optimize the attachment of composite grafts in the reconstruction of small complex facial defects by combining it with Fluorescent Light Energy (FLE) during the healing process of the graft. The beneficial effects of photobiomodulation (PBM) on wound healing might be attributed to anti-inflammatory signaling, cell proliferation, protein synthesis, and decreased bacterial infection. We previously experienced rewarding achievements using Photonic energy in the treatment of burns, non-healing wounds and pathological scars. Therefore, we chose to exploit the potential of bioluminescent energy to maximize aesthetical and functional results, enhancing the formation of new vascular connections and modulating both inflammatory and scarring processes. From the 2nd postoperative day, the patients were locally treated with 5 FLE sessions every (48/72 h) 2 or 3 days. We evaluated results in terms of time for engraftment, quality of the scarring, infective complications and morbidity of the donor site. Graft survival is subject to many factors, both local and systemic. To overcome these issues, various methods have been studied and described. We here report the successful reconstruction of full-thickness defects of the free margin of the nasal alar rim and the central portion of the inferior lip with a composite graft. These results were highly encouraging if compared with the literature. By analyzing our small cohort, we demonstrated how new technologies may push this traditional reconstructive procedure beyond their old boundaries: overcoming an increased size demand or a potential infective wound environment.

## Introduction

Single-stage repair of small complex facial defects still represents a challenge for the reconstructive surgeon. The free composite graft technique was first introduced by Flanagin in 1956 [1] and has assumed a more and more relevant role in the restoration of specific aesthetic subunits.

Facial soft tissue subunit defects are technically challenging to reconstruct due to variable skin texture, contour, and color. Surgical reconstruction requires a delicate balance between reshaping the nasal curvatures and predicting scar contracture [2,3].

Several techniques have been proposed for the reconstruction of the nasal ala and the soft triangle including skin grafts, composite grafts and various local flaps. None of these techniques are currently considered as the golden standard. Excellent results may be reached with a composite full-thickness graft from the ear [4].

For what concerns the lips, they play a dynamic role in facial esthetics, human communications, and oral functions. Full-thickness defects require meticulous attention to obtain a three-layer closure of mucosa, muscle, and skin [5]. Many different reconstructive solutions have been suggested for lip reconstruction including skin grafts, composite grafts, local flaps and free flaps. After its introduction Millard, as well as many surgeons, applied the free composite graft technique for secondary repair of cleft lip deformity [6–9].

Composite grafts have high metabolic demands. The traditional recommendation is to limit the size of composite grafts to 1 cm or less due to limited blood flow, which otherwise will lead to tissue necrosis of the graft [4,10–12].

Main limitations include variability and unpredictability of ultimate graft survival.

It should not be designed larger than 2 cm in its greatest dimension and not be performed in smokers and patients with small-vessel disease, such as diabetes or coronary artery disease.

Skouge [13] advocated a tongue-in-groove technique when using composite grafts. This technique involves harvesting the graft with cartilage struts extending beyond the borders of the soft tissue and inserting the border of the graft between 2 layers of tissue at the recipient site.

Other adjunctive measures to increase the intake of a composite graft include surface cooling with iced compresses, which decreases the metabolic demand of the graft; hyperbaric oxygen therapy, which promotes fibroblast replication, collagen formation, and neovascularization; and pre and postoperative corticosteroids [10]. Finally, scarification with a thin needle (20–26 gauge) coupled with heparin injections can be used for decongestion of the graft [14].

Research is looking for new alternatives which could improve the engraftment, widen the indications and ease the replicability.

Fluorescent light energy (FLE) therapy modulates biological processes in different tissues, with a positive effect on many cell types and pathways essential for wound healing [15].

The beneficial effects of Photobiomodulation (PBM) on wound healing might be attributed to anti-inflammatory signaling, cell proliferation, protein synthesis, and decreased bacterial infection [16].

The BioPhotonic gel, known as LumiHeal™ (KLOX Technologies Inc., Laval, QC, Canada) consists of a gel containing light-absorbing molecules (chromophores) that are illuminated by a multi-LED lamp.

Chromophores capture light emitted by a blue LED lamp and then convert it in a wider emission visible spectrum (fluorescence).

The wavelength range is 532–615 nm. Several studies have shown that the most effective wavelengths in penetrating the skin and promoting healing are those of blue, green, yellow, and orange; each wavelength corresponds to a precise biological effect (Table 1).

**Table 1.** Wavelengths of FLET and their biological effects.

Visible light spectrum effects on biological targets				
Wavelength range	Visible light spectrum	Biological effect	Target	Main outcome
660–780 nm	Yellow to Red	Decrease in fluid loss, tissue destruction and inflammation while increasing the deposition of collagen fibers, larger amounts of granulation tissue, less edema, a more vigorous inflammatory reaction and increased revascularization	Third-degree burns in rats	Inflammatory Modulation
632.8-nm	Yellow to Orange	Significantly increases gene expression of TGF-β1, while significantly reducing inflammation in both nondiabetic and control rats leading to wound healing	Surgically tenotomized Achilles tendons in rats	Inflammatory Modulation
660 and 880 nm	Red	Reduces the initial ulcer area by 13-fold versus control subjects	Patients with grade II pressure ulcers	Tissue proliferation and matrix deposition
660 nm	Red	Full granulation and ulcer closure in 86% of the patients	Patients with recalcitrant venous ulcers due to chronic venous insufficiency syndrome	Tissue proliferation and matrix deposition
633 or 830 nm	Yellow to Red	Cell migration, viability and proliferation	Skin fibroblasts in diabetic wounds	Tissue proliferation and matrix deposition
633 nm	Yellow/Orange	Alleviating induced rat diabetic ulcers by an average of about 10%, although some diabetic ulcers can achieve 39% improvements	Diabetic ulcers	Tissue proliferation and matrix deposition
632.8	Red	Tissue healing by inducing a significant increase in dermal angiogenesis	Chronic radiation-induced dermatitis in breast cancer	Tissue proliferation and matrix deposition
670 nm	Red	Human umbilical vein endothelial cell proliferation	Neoangiogenesis and wound healing	Neoangiogenesis and wound healing
660 nm	Red	Increased secretion of the growth factors VEGF, basic FGF and HGF as well as increased neovascularization of the wound bed and regeneration of the skin than occurs in controls	Skin wound bed in athymic mice	Neoangiogenesis
400–800 nm	Violet to Red (full range)	62 and 83%, and 56% reduction in the colony count of E. coli, Streptococcus aureus and S. marcescens, respectively	Bacterial strains from clinical specimens	Antibacterial actions
530 nm	Green	Inactivating bacteria such as S. mutans that is related to dental caries	Cariogenic bacteria	Antibacterial actions

Created by the authors with the following references. 1. Meirelles GC, Santos JN, Chagas PO, et al. A comparative study of the effects of laser photobiomodulation on the healing of third-degree burns: a histological study in rats. Photomed Laser Surg. 2008;26(2), 159–166. 2. Aliodoust M, Bayat M, Jalili MR, et al. Evaluating the effect of low-level laser therapy on healing of tenotomized Achilles tendon in streptozotocin-induced diabetic rats by light microscopical and gene expression examinations. Lasers Med Sci. 2014;29(4):1495–1503. 3. Dehlin O, Elmstahl S, Gottrup F. Monochromatic phototherapy: effective treatment for grade II chronic pressure ulcers in elderly patients. Aging Clin Exp Res. 2007;19(6):478–483. 4. Dixit S, Maiya AG, Umakanth S, et al. Closure of non-healing chronic ulcer in Klippel-Trenaunay syndrome using low-level laser therapy. BMJ Case Rep. 2012;2012:bcr2012006226. doi:10.1136/bcr-2012-006226. 5. Houreld N, Abrahamse H. Low-intensity laser irradiation stimulates wound healing in diabetic wounded fibroblast cells (WS1). Diabetes Technol Ther. 2010;12(12):971–978. 6. Al-Watban FA. Laser therapy converts diabetic wound healing to normal healing. Photomed Laser Surg. 2009;27(1):127–135. 7. Schindl A, Schindl M, Pernerstorfer-Schon H, et al. Low intensity laser irradiation in the treatment of recalcitrant radiation ulcers in patients with breast cancer – long-term results of 3 cases. Photodermatol Photoimmunol Photomed. 2000;16(1):34–37. 8. Schindl A, Merwald H, Schindl L, et al. Direct stimulatory effect of low-intensity 670 nm laser irradiation on human endothelial cell proliferation. Br J Dermatol. 2003;148(2):334–336. 9. Park IS, Chung PS, Ahn JC. Adipose-derived stromal cell cluster with light therapy enhances angiogenesis and skin wound healing in mice. Biochem Biophys Res Commun. 462(3):171–177 (2015). 10. Lipovsky A, Nitzan Y, Lubart R. A possible mechanism for visible light-induced wound healing. Lasers Surg Med. 2008; 40(7):509–514. doi: 10.1002/lsm.20668. 11. Nagata JY, Hioka N, Kimura E, et al. Antibacterial photodynamic therapy for dental caries: evaluation of the photosensitizers used and light source properties. Photodiagnosis Photodyn Ther. 2012;9(2):122–131.

Wavelengths in the 500–700 nm range are suitable for treating superficial tissue, while wavelengths between 800 and 1000 nm are suitable for deeper tissues [17].

FLE Systems comprise a multi-LED lamp (KT-L lamp, Klox Technologies Inc., Laval, QC, Canada) and a topical photoconverter substrate available as an amorphous gel (FLE-Gel) or sheet hydrogel matrix (FLE-Matrix) (LumiHealTM Gel and LumiHealTM Matrix, Klox Technologies Inc., Laval, QC, Canada). The multi-LED lamp emits non-coherent light in the 400–520 nm range, peaking at approximately 447 nm, with a power density between 110–150 mW/cm^2^ at a 5 cm distance from the LEDs. The lamp features a 5-minute timer and a distance indicator. The FLE photoconverters contain a chromophore within the gel or matrix that absorbs some photons from the multi-LED lamp and emits FLE in the 510–700 nm range. Consequently, cells treated with FLE are exposed to both direct light from the multi-LED lamp and FLE emitted from the Gel or Matrix photoconverter, covering a spectral range of 400–700 nm. Notably, a dose response for FLE can be observed by comparing FLE-Gel and FLE-Matrix, as FLE-Gel produces 0.1–0.2 J/cm^2^ of fluorescence (approximately 510–700 nm) whereas FLE-Matrix produces 0.2–0.7 J/cm^2^ [18].

We performed three composite grafts: two in an elective setting for alar nasal reconstruction using a chondrocutaneous flap from the auricular helix, and one in an emergency setting for labial reconstruction following a dog bite injury. Revascularization of the grafts begins between 24 and 48 h post-attachment. Therefore, our protocol, aimed at accelerating the neoangiogenesis process, starts on the second postoperative day. Patients undergo five FLET sessions every 2 or 3 days.

The procedure was performed under general anesthesia. Sterile draping and iodopovidone disinfection are used for each case. Preoperative single dose of 2 g of Cefazolin was administered. All defect areas were thoroughly debrided and irrigated. In cases where the composite graft site is potentially contaminated (such as in emergency settings), thorough debridement is conducted to remove any debris and necrotic tissue, followed by multiple pressure washings with physiological saline solution. After the surgical procedure the dressing is done with paraffinated gauze and sterile gauzes.

At 48 h, after meticulous removal of the dressing, we performed the FLET treatment. To prepare the chromophore solution, it must be gently mixed with the inert gel to obtain a homogeneous preparation, avoiding prolonged light exposure. After proper disinfection and cleansing with saline solution, the chromophore gel is then applied to the graft area and the surrounding skin, initiating the photonic therapy. The LED lamp is positioned 5 cm from the skin surface in a dark room environment, and the treatment lasts for 5 min. During the procedure, all patients, physicians, and caregivers exposed to the lamp light wear protective glasses. Our protocol includes 3–5 sessions, with each treatment performed every 48 to 72 h If any pain, burning sensation, or allergic response is suspected, the treatment should be immediately suspended, and the area thoroughly cleansed. No reactions or side effects were observed during our study (Table 2).

## Results

All the patients were treated with the same protocol as previously described (Figures 1–14).

**Figure 1.**
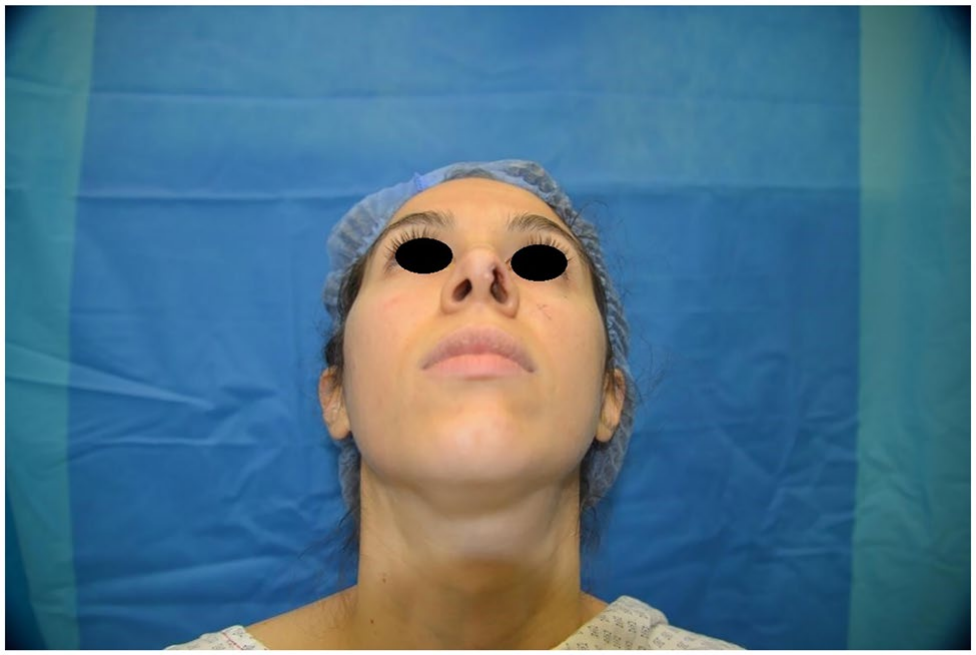
Preoperative picture. Property of plastic surgery unit, University Hospital of Padova, Padova, Italy. The patient provided informed consent and authorized the use of the picture for all scientific purposes.

**Figure 2.**
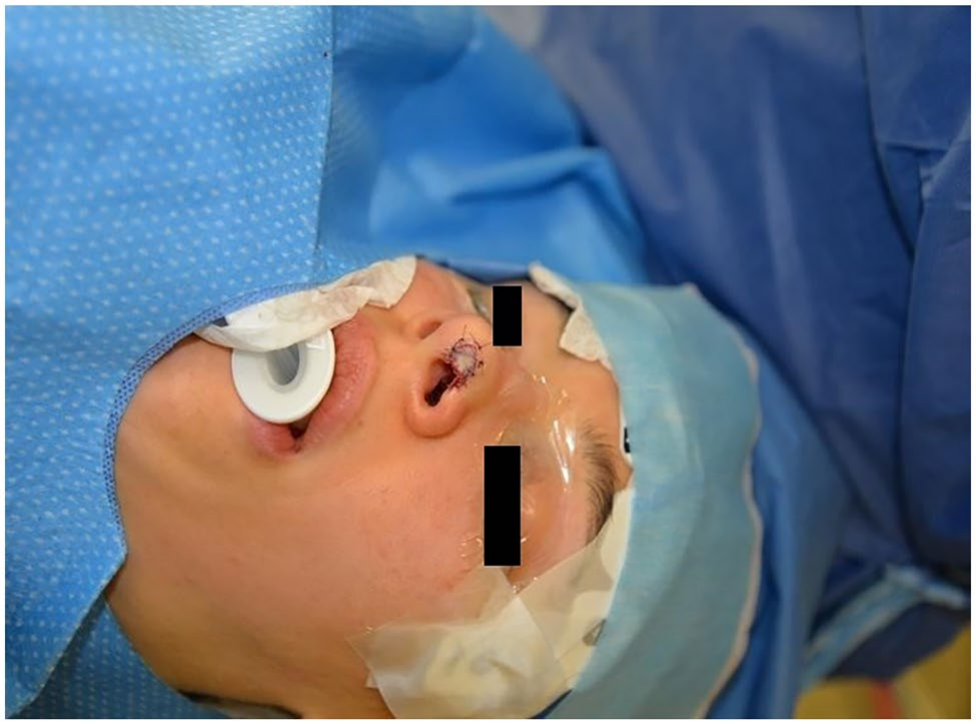
Intraoperative picture. Property of plastic surgery unit, University Hospital of Padova, Padova, Italy. The patient provided informed consent and authorized the use of the picture for all scientific purposes.

**Figure 3.**
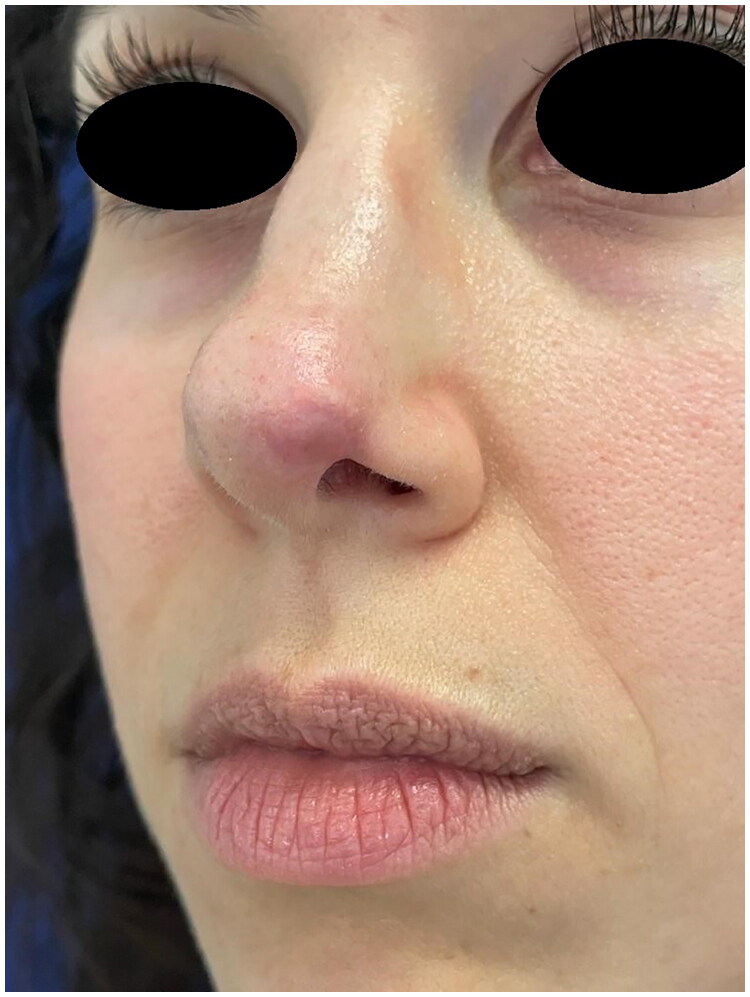
4 Weeks postoperative picture. Property of plastic surgery unit, University Hospital of Padova, Padova, Italy. The patient provided informed consent and authorized the use of the picture for all scientific purposes.

**Figure 4.**
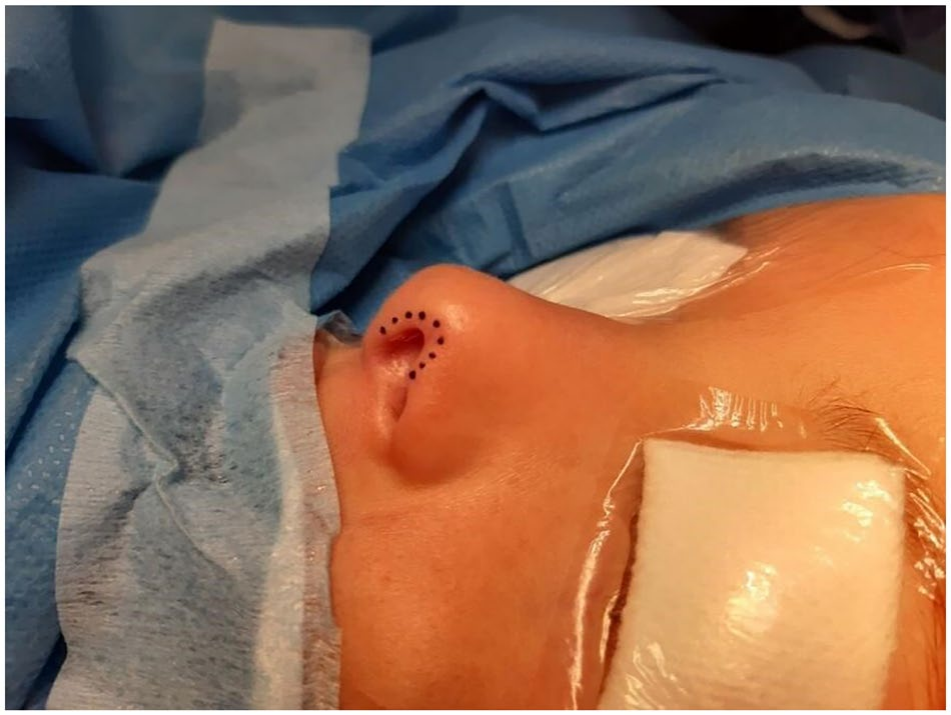
Pre-operative marking: defect. Property of plastic surgery unit, University Hospital of Padova, Padova, Italy. The patient provided informed consent and authorized the use of the picture for all scientific purposes.

**Figure 5.**
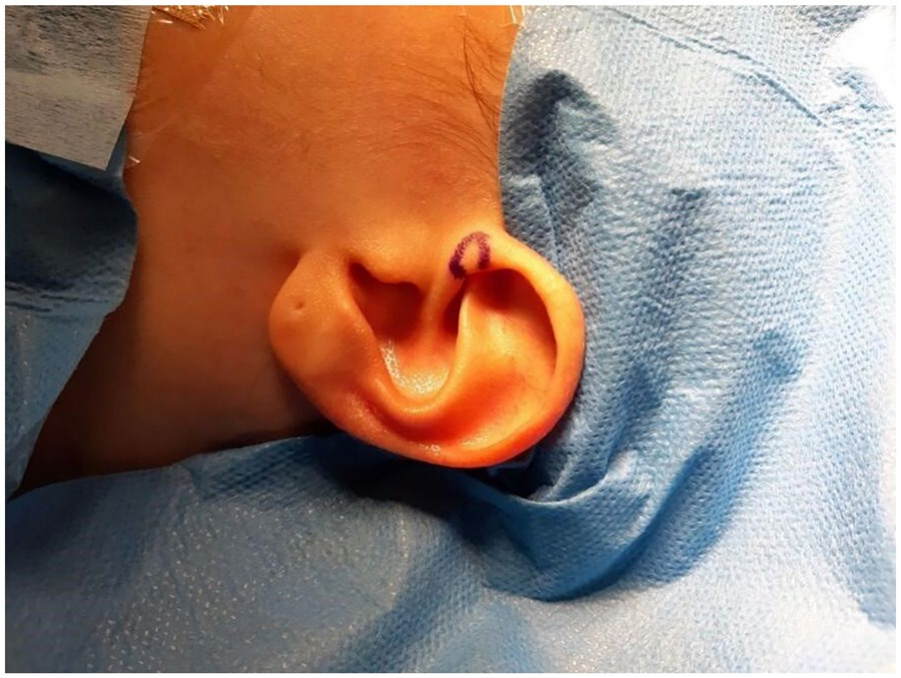
Pre-operative marking: donor graft site.

**Figure 6.**
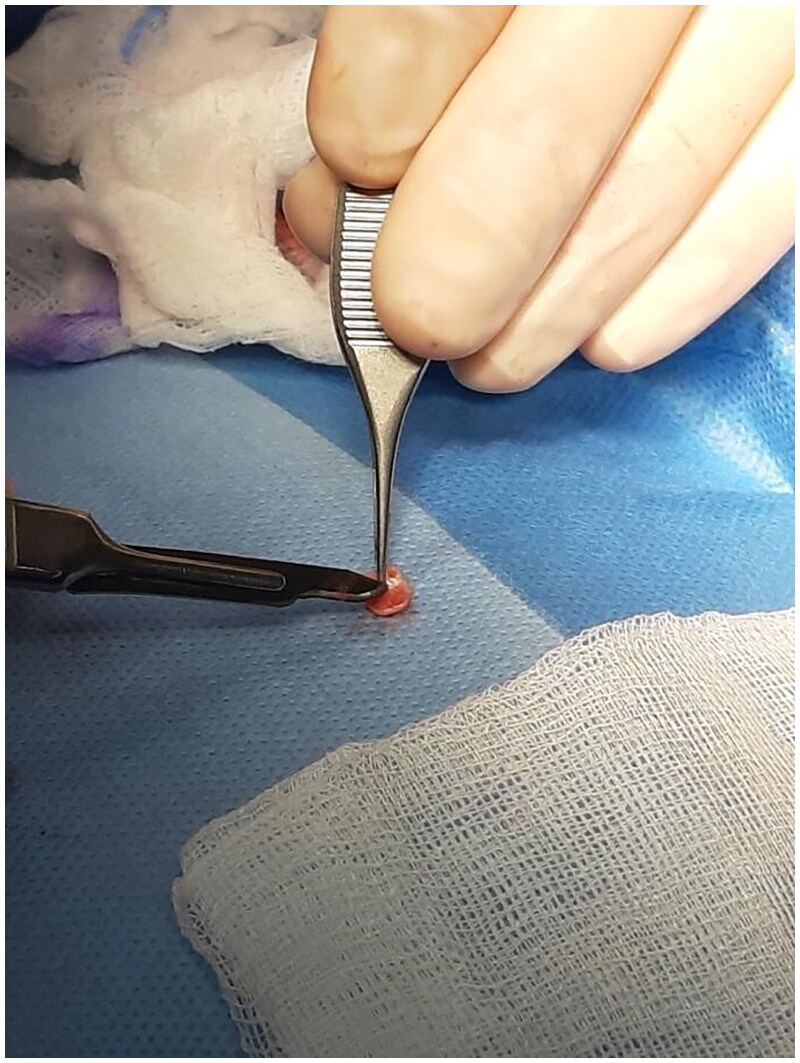
Shaping of the graft. Property of plastic surgery unit, University Hospital of Padova, Padova, Italy. The patient provided informed consent and authorized the use of the picture for all scientific purposes.

**Figure 7.**
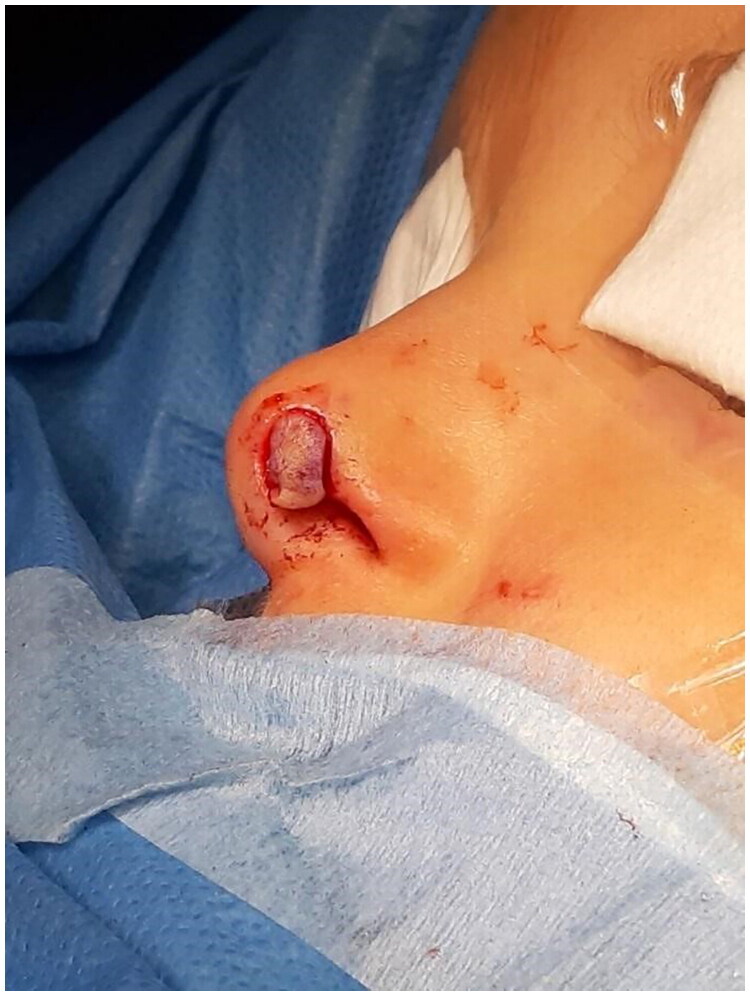
Intraoperative: attachment of the graft. Property of plastic surgery unit, University Hospital of Padova, Padova, Italy. The patient provided informed consent and authorized the use of the picture for all scientific purposes.

**Figure 8.**
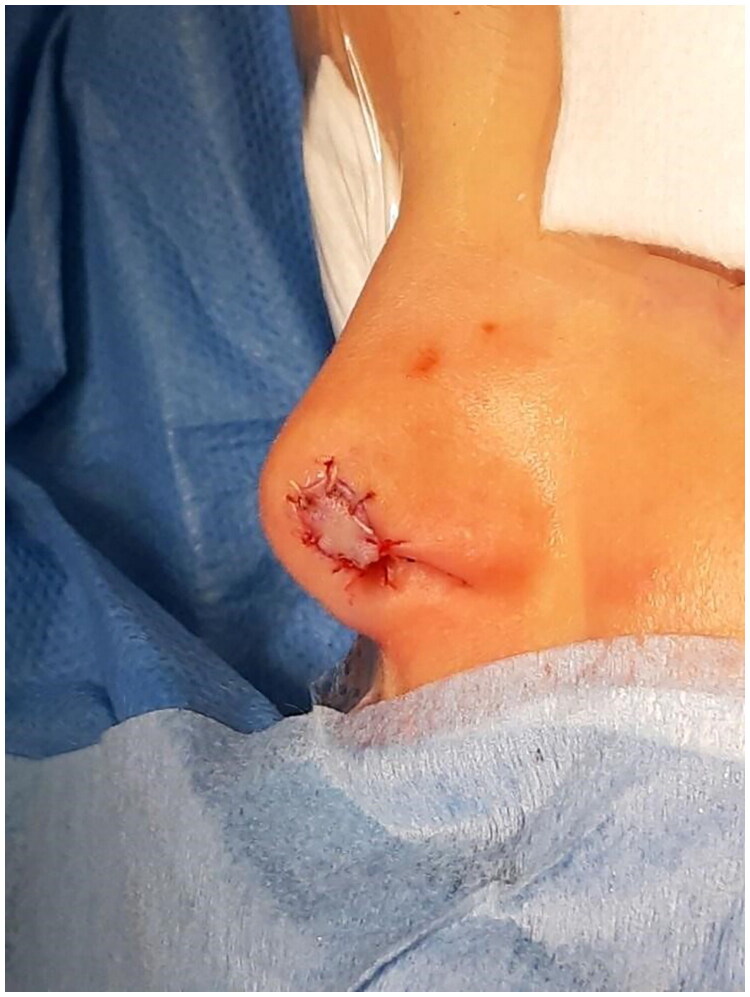
Intraoperative: attachment of the graft. Property of plastic surgery unit, University Hospital of Padova, Padova, Italy. The patient provided informed consent and authorized the use of the picture for all scientific purposes.

**Figure 9.**
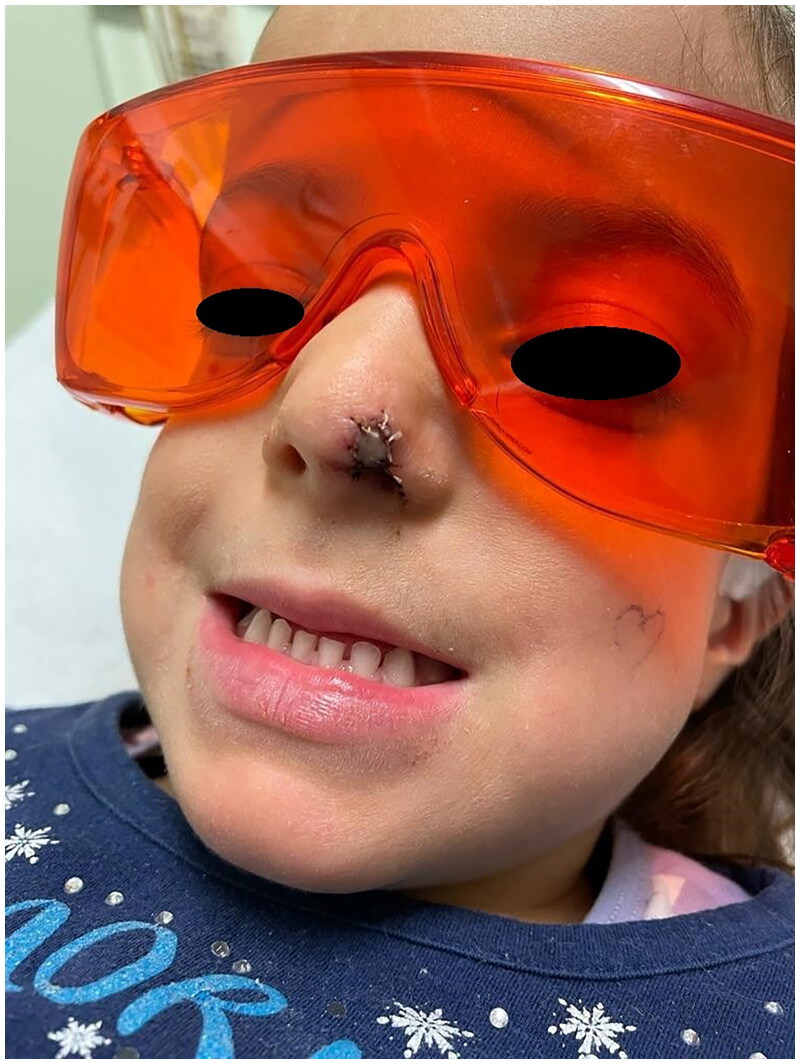
4 Days postoperative picture: during FLET therapy. Property of plastic surgery unit, University Hospital of Padova, Padova, Italy. The patient provided informed consent and authorized the use of the picture for all scientific purposes.

**Figure 10.**
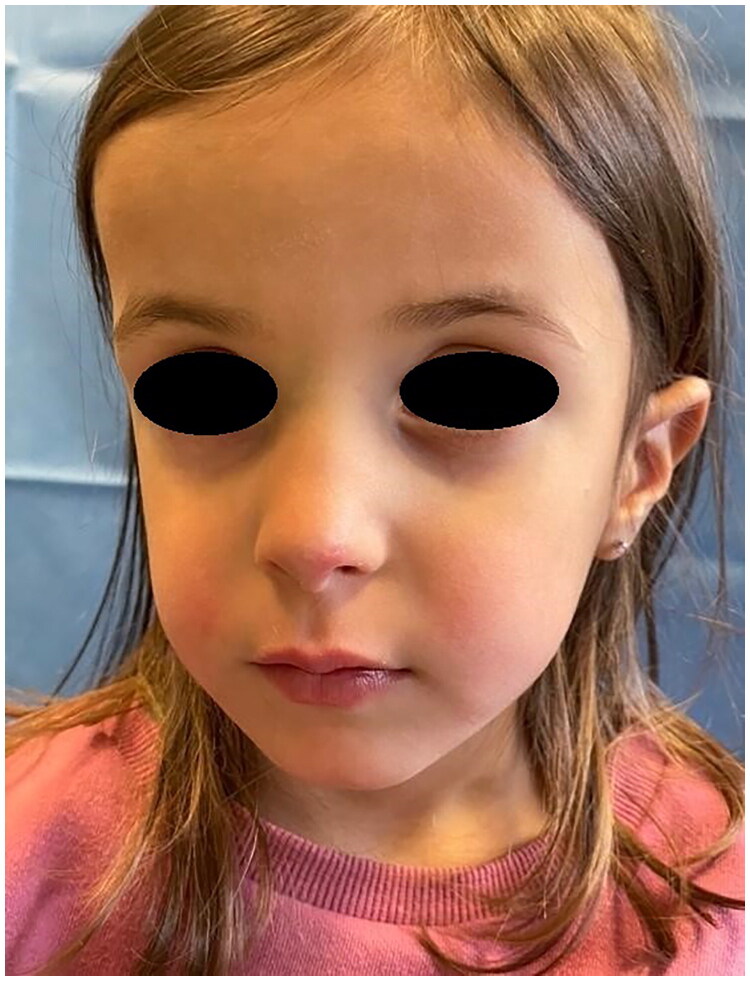
15 Days postoperative picture property of plastic surgery unit, University Hospital of Padova, Padova, Italy. The patient provided informed consent and authorized the use of the picture for all scientific purposes.

**Figure 11.**
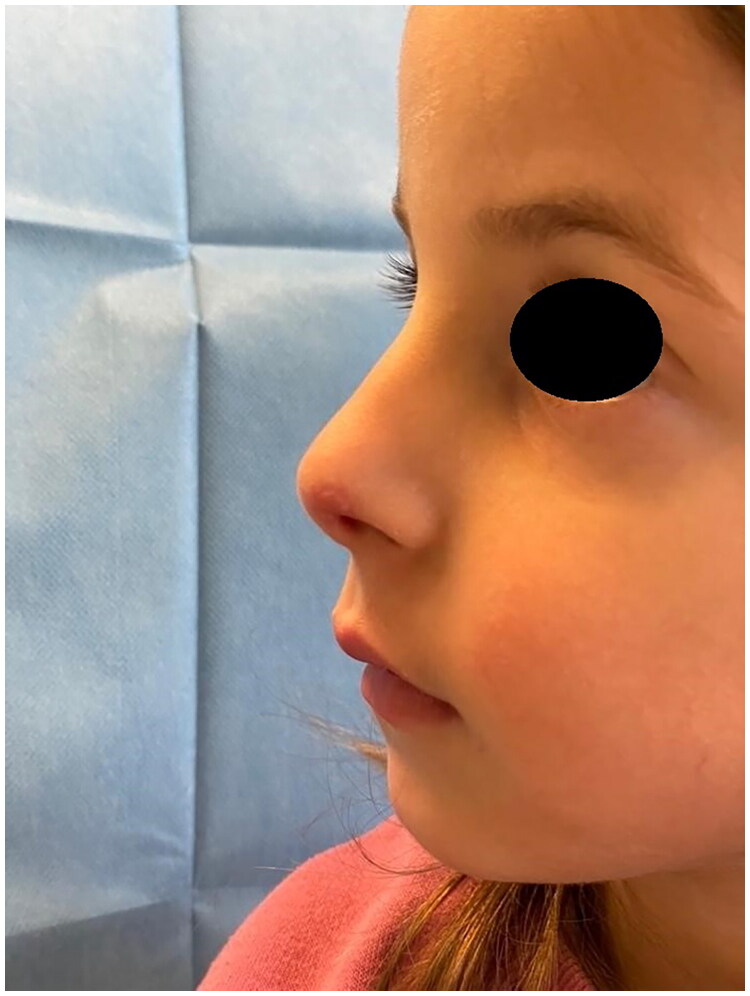
15 Days postoperative picture. Property of plastic surgery unit, University Hospital of Padova, Padova, Italy. The patient provided informed consent and authorized the use of the picture for all scientific purposes.

**Figure 12.**
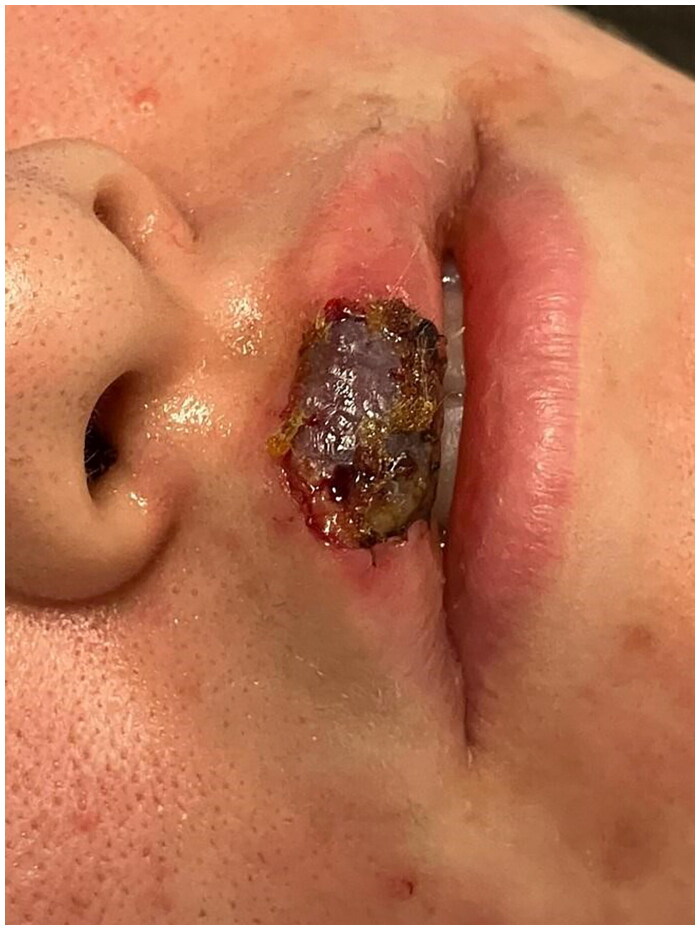
3 Days postoperative picture. Property of plastic surgery unit, University Hospital of Padova, Padova, Italy. The patient provided informed consent and authorized the use of the picture for all scientific purposes.

**Figure 13.**
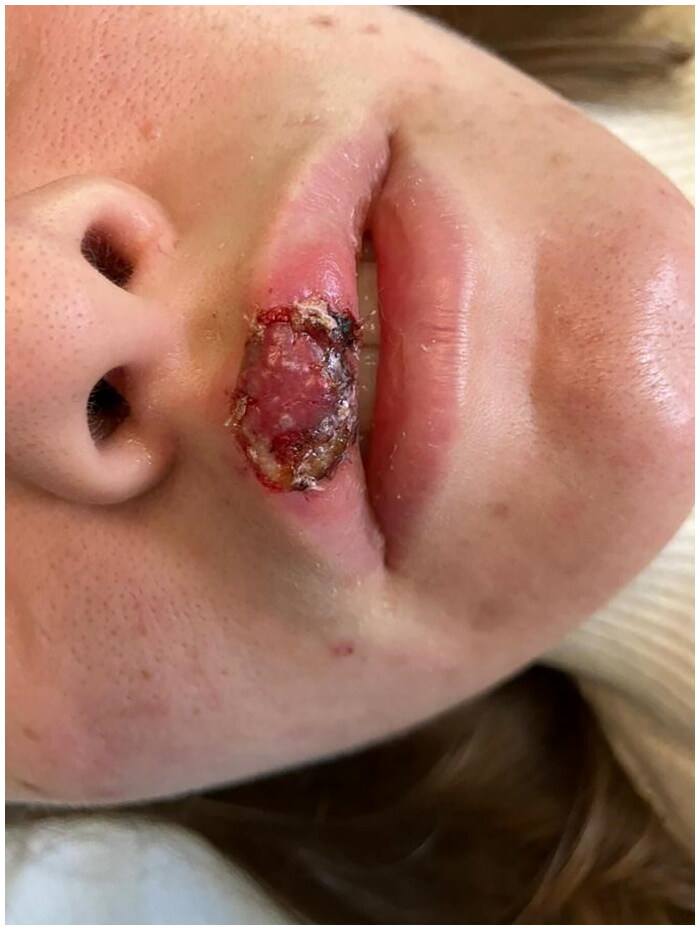
3 Days postoperative after FLET treatment. Property of plastic surgery unit, University Hospital of Padova, Padova, Italy. The patient provided informed consent and authorized the use of the picture for all scientific purposes.

**Figure 14.**
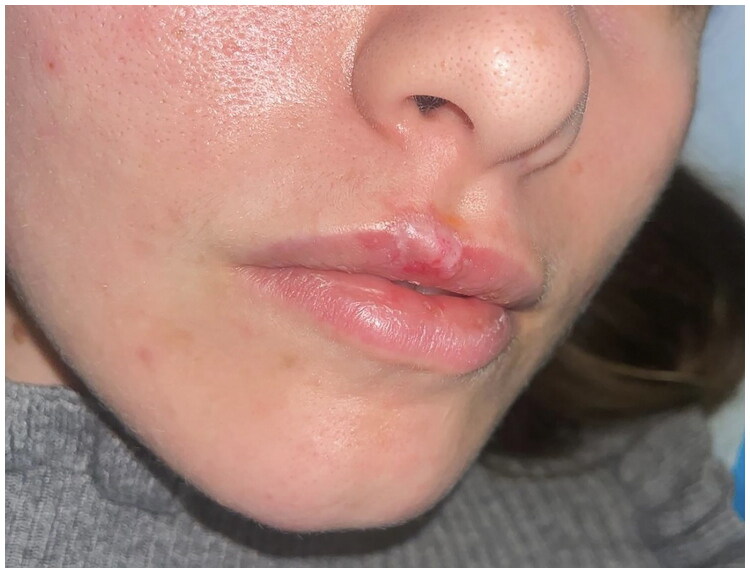
4 Weeks postoperative picture. Property of plastic surgery unit, University Hospital of Padova, Padova, Italy. The patient provided informed consent and authorized the use of the picture for all scientific purposes.

### Case 1:

A 30 y.o. female patients with left nasal alar defect as consequence of a dog bite. Both alar cartilage and the soft triangle layer were involved. 10 days after the trauma, following an appropriate antibiotic therapy (amoxicillin and clavulanic acid pot 875 mg/125 mg three times a day for 6 days) and local dressings, the patient underwent the reconstructive surgery through a composite condro-cutaneous graft harvested from the left auricular helix. The graft was fixed after an appropriate debridement to the wound margins with 5.0 Vicryl Rapid and 5.0. Nylon. Dressing was done with paraffined gauze and sterile gauzes.

The donor site was repaired with direct suture with 5.0. Nylon.

During the healing phase the patient underwent 5 FLE sessions every 3 days since the 2th postoperative day.

### Case 2:

A 5 y.o. child presenting a full-thickness left alar pressure wound deformity due to prolonged nasogastric intubation.

The procedure included the dissection of the condro-cutaneous flap of the ala which was overturned.

The graft, which was raised from the left radix of the helical root, was then shaped, thinned and placed over the flap to recreate the natural thickness and curvature of the soft tissue triangle. The graft was fixed with 5.0. Vicryl Rapid stitches while the ear was closed with direct suture in 4.0. Vicryl and 5.0. Vicryl Rapid stitches.

From the 2nd postoperative day the patient was locally treated with 5 FLE sessions every 2 days.

#### Case 3:

A 23 y.o. female patient presented to the ER for a dog bite which had ripped her off a full thickness flap of inferior labial mucosae, subcutaneous and orbicularis oris muscle.

After appropriate irrigation and debridement of the wound bed, the torned part of the lip was shaped and used as a composite graft and was sutured with 4.0. Vicryl Rapid stitches.

During the attachment process, the patient underwent systemic antibiotic treatment (amoxicillin and clavulanic acid pot 875 mg/125 mg three times a day for 6 days), and as per protocol we implemented from 2th post-operative day 5 FLE sessions every 3 days.

## Discussion

For the reconstruction of small specific areas for post-oncological, post-traumatic or malformative reasons the composite graft, both heterotopic and homotopic, represents an ancillary single-stage procedure that can offer satisfying outcomes.

Especially, when a rigid structure with a soft tissue coverage or a multi-layered myocutaneous segment is required, composite grafting may offer an ideal solution. This technique is reportedly still underused in facial reconstructive surgery due to high rates of graft failure which are referable to blood supply issues [19].

The imbibition of the graft and neo angiogenesis remain the crucial elements for its survival and that explains the efforts of various authors to improve and ease these processes. Metabolic requests grow with the size and vary with the composition of the graft.

Literature agrees with dimensional safety boundaries of 2–3 cm width defects to be repaired with composite grafting. Smoking, Vessels disease and infective settings are still considered as main contraindications for this procedure [12,20,21].

Different maneuvers, as previously described, have been suggested to establish a stable and early blood supply to the graft intake. Recognized key factors are the shrewdness in the graft design and an appropriate preparation of the defect site [10,14,20].

The manipulation of the graft and cartilage remains a topic of debate. On one hand, cartilage scarification is believed to stimulate chondroblast regeneration, but traumatizing such small grafts may compromise their integrity and increase the risk of damage [22].

The classic Hirase technique [23], which incorporates cold packs and aluminum foils for composite grafts in distal replantation of fingertip avulsions, aims to lower the temperature of the amputated finger to reduce metabolic demands for the first 72 h. This approach minimizes cellular degradation until neoangiogenesis begins. However, applying this principle to anatomical areas like the nose and lip is impractical, as using ice in these regions is challenging. Moreover, improper contact with cold sources can result in frostbite and graft failure. We believe that accelerating angiogenesis and natural healing processes in the graft is preferable to merely reducing its metabolic demands, giving FLET a significant advantage.

Some authors advocate for the use of local anticoagulants to prevent vascular congestion and promote metabolic exchanges in the graft. However, the increased risk of bleeding can lead to hematomas and subsequent graft loss. Steroids, known for modulating scarring processes, can cause tissue atrophy and delayed healing. Additionally, the topical treatments mentioned, such as cold packing, heparin, and steroid injections, are often painful and poorly tolerated in the facial area, especially by younger patients.

While hyperbaric therapy can enhance cellular metabolism and promote neoangiogenesis, it requires multiple long sessions in a closed environment, and the necessary equipment is not widely available in smaller centers.

Although these techniques have theoretical merit, they may not be suitable for all patients and are often difficult to reproduce consistently. The simplicity and accessibility of FLET treatment may eliminate the need for these methods while being equally effective, if not superior.

We investigated new technologies for something which could improve blood supply while acting on the scarring process as secondary soft tissue contracture can lead to a very poor cosmetic outcome [19].

Our practice and scientific reports on PBM, especially FLE KLOX technology in the management of wounds showed its ease of use and its low dependence on the operator [19,24].

Absorption of photons by CCO (complex IV of the mitochondrial respiratory chain, cytochrome c oxidase) initiates a biochemical cascade that increases ATP and ROS generation within the electron transport chain [25] FLE is a unique form of photobiomodulation that has been demonstrated to enhance the healing of both acute and chronic wounds [26]. The beneficial impact of FLE on inflammation has been well documented, with improved inflammatory profiles observed in clinical trials for wound healing [27], acne treatment [28], and management of rosacea [29] and mechanistic *in vitro* studies [30]. These studies have demonstrated that FLE can reduce pro-inflammatory cytokines produced by human dermal fibroblasts (HDFs), as well as increase levels of some anti-inflammatory cytokines, which positively impact the treatment of chronic wounds.

A series of case studies investigating the effect of FLE treatment on acute second-degree burns demonstrated accelerated wound healing and overall improvement of tissue structure in two cases of severe hypertrophic scars (HTS) after burn injuries, suggesting that FLE balances wound healing at different stages of the wound healing and remodeling process [31]. Since angiogenesis is vital for wound healing, we evaluated the number of blood vessels in the collected xenografts. Angiogenesis is crucial for restoring circulation in grafted and damaged tissue and is induced by growth factors such as vascular endothelial growth factor (VEGF) produced by keratinocytes, fibroblasts, and macrophages [32].

We found that FLE treatment stimulated healing by increasing re-epithelialization, significantly increasing epidermal thickness, and reducing dermal thickness after 28 days, while decreasing the number of blood vessels after 56 days. Moreover, collagen production was enhanced at day 28 and significantly reduced at day 56, with collagen orientation index (COI) significantly reduced at all three time points. Finally, mast cell infiltration, myofibroblast formation, and angiogenesis were lowered later in the healing process (84 days post-treatment) [33].

Generally, we reported very high tolerance to the treatment representing an optimal choice for children and fragile patients. Encouraging non-clinical experimental results by Ding et al. enlightened how Fluorescent light energy modulates healing in skin grafted mouse model. They demonstrated how FLE may be effective in the promotion of angiogenesis and collagen production but capable of limiting the undesirable overexpression of VEGF-A and normalizing collagen deposition and remodeling in the tissue in the long-term [15].

We speculated that FLE could offer a contribution in terms of graft take and aesthetic outcome when composite grafting is used, this improved healing could potentially overcome the traditional dimensional limits of the composite grafts.

In the 3rd case, we applied this technology to gain both an anti-inflammatory and antiseptic action in the replantation of the avulsed composite tissue obtaining a promising post-procedural result.

## Conclusions

In conclusion, we successfully reconstructed full-thickness defects of the free margin of the nasal alar rim and the central portion of the superior lip using composite grafts. Our outcomes demonstrated excellent functional and cosmetic results, even in high-risk infective settings.

Future studies are needed to elucidate the specific molecular pathways activated by FLE treatment that contribute to its beneficial effects. Potential mechanisms likely involve photon absorption by endogenous chromophores, utilization of photonic energy, and modulation of mitochondrial activity, including ATP production. These processes regulate cellular activation, migration, and protein synthesis, which are critical in wound healing. However, the small number of cases in our study limits the generalizability of our findings.

We believe that surgeons should consider FLE as a valuable tool to enhance composite graft survival and healing and improve scar process. Expanding the use of FLE in clinical practice could significantly benefit patient outcomes in reconstructive surgeries.

**Table 2.** Protocol and treatment session details.

Parameter	Details
Initiation timing	From 48 h post-operation
Distance from target	=/5 cm
Duration	5 min
Frequency	every 2 or 3 days
Number of sessions	3–5
Cessation criteria	Non compliance, pain, burning sensation or allergic response

Property of plastic Surgery unit, University Hospital of Padova *via* Giustiniani 2, 35128, Padova, Italy.

## References

[1] Flanagin WS. Free composite grafts from lower to ­upper. Plast Reconstr Surg. 1956;17(5):376–380. doi: 10.1097/00006534-195605000-00005. 13335514

[2] Goldman A, Wollina U, et al. Dog bite injury—alar ­repair with composite graft. Wien Med Wochenschr. 2016;168:261–264. doi: 10.1007/s10354-016-0523-5. 27807675

[3] Therattil PJ, Kass WS, Sood A, et al. Alar rim reconstruction: a case report and review of the literature. Madridge J Case Rep Stud. 2017;1(1):11–15. doi: 10.18689/mjcrs-1000103.

[4] Son D, Kwak M, Yun S, et al. Large auricular chondrocutaneous composite graft for nasal alar and columellar reconstruction. Arch Plast Surg. 2012;39(4):323–328. doi: 10.5999/aps.2012.39.4.323. 22872834 PMC3408276

[5] Baumann D, Robb G. Lip reconstruction. Semin Plast Surg. 2008;22(4):269–280. PMID: 20567703; PMCID: PMC2884873.] doi: 10.1055/s-0028-1095886. 20567703 PMC2884873

[6] Davenport G, Bernard FD. Improving the take of composite grafts. Plast Reconstr Surg Transplant Bull. 1959;24(2):175–182. doi: 10.1097/00006534-195908000-00004. 13814248

[7] Millard DR. Composite lip flaps and grafts in secondary cleft deformities. Br J Plast Surg. 1964;17:22–29. doi: 10.1016/s0007-1226(64)80005-9. 14103349

[8] Moore FT, Lendvay PG. Free compositelip-switch procedure. Br J Plast Surg. 1969;22:261–264. 10.1016/s0007-1226(69)80116-5 5387146

[9] Soeda S. Free compositegraft of the lip. Experimental study of revascularization and clinical application. J Jpn Plast Reconstr Surg. 1975;18:289–301.

[10] Kim G, Jeong Y-I, Shim H-C, et al. Auricular composite chondrocutaneous grafts in the repair of nasal alar rim defects. Ann Dermatol. 2014;26(3):407–408. doi: 10.5021/ad.2014.26.3.407. 24966648 PMC4069659

[11] van der Eerden PA, Verdam FJ, Dennis SCR, et al. Free cartilage grafts and healing by secondary intention: a viable reconstructive combination after excision of nonmelanoma skin cancer in the nasal alar region. Arch Facial Plast Surg. 2009;11(1):18–23. doi: 10.1001/archfacial.2008.501. 19153288

[12] Son D, Jeong H, Choi T, et al. A new mechanism associated with composite graft success. J Plast Reconstr Aesthet Surg. 2010;63(11):1900–1909. doi: 10.1016/j.bjps.2009.11.018. 20036629

[13] Skouge JW. Skin grafting. New York: Churchill Livingstone; 1991.

[14] Cherpelis BS, Carls JL. One-stage reconstruction of a full-thickness nasal defect involving the alar rim. Dermatol Surg. 2007;33(11):1361–1364. doi: 10.1111/j.1524-4725.2007.33292.x. 17958592

[15] Ding J, Mellergaard M, Zhu Z, et al. Fluorescent light energy modulates healing in skin grafted mouse model. Open Med. 2021;16:1240–1255. 10.1515/med-2021-0329 PMC8402934 34522783

[16] de Freitas LF, Hamblin MR. Proposed mechanisms of photobiomodulation or low-level light therapy. IEEE J Sel Top Quantum Electron. 2016;22(3):348–364. pii doi: 10.1109/JSTQE.2016.2561201. PMC5215870 28070154

[17] Kanai A. Xenon light therapy. Masui. 2012;61(7):693–699. 22860297

[18] Ferroni L, Zago M, Patergnani S, et al. Fluorescent light energy (FLE) acts on mitochondrial physiology improving wound healing. J Clin Med. 2020;9(2):559. PMID: 32085605; PMCID: PMC7073965.] doi: 10.3390/jcm9020559. 32085605 PMC7073965

[19] Yamasaki A, Dermody SM, Moyer JS. Reducing risks of graft failure for composite skin-cartilage grafts. Facial Plast Surg Clin North Am. 2023;31(2):289–296. ] doi: 10.1016/j.fsc.2023.01.007. 37001931

[20] Teltzrow T, Arens A, Schwipper V. One-stage reconstruction of nasal defects: evaluation of the use of modified auricular composite grafts. Facial Plast Surg. 2011;27(3):243–248. ] doi: 10.1055/s-0031-1275773. 21567343

[21] Tomioka Y, Okazaki M, Kurita M, et al. Stair-step incision for composite grafts in nasal reconstruction. J Craniofac Surg. 2023;34(8):2464–2467. doi: 10.1097/SCS.0000000000009476. 37316982

[22] Silver FH, Glasgold AI. Cartilage wound healing. An overview. Otolaryngol Clin North Am. 1995;28(5):847–864. doi: 10.1016/S0030-6665(20)30463-1. 8559576

[23] Adani R, Marcoccio I, Tarallo L. Treatment of fingertips amputation using the Hirase technique. Hand Surg. 2003 Dec;8(2):257–264. PMID: 15002108.] doi: 10.1142/s0218810403001777. 15002108

[24] Edge D, Mellergaard M, Dam-Hansen C, et al. Fluorescent light energy: the future for treating inflammatory skin conditions? J Clin Aesthet Dermatol. 2019;12(5):E61–E68. ] PMC6561711 31320979

[25] Romanelli M, Piaggesi A, Scapagnini G, et al. Evaluation of fluorescence biomodulation in the real-life management of chronic wounds: the EURIKA trial. J Wound Care. 2018;27(11):744–753. doi: 10.12968/jowc.2018.27.11.744. 30398941

[26] Mosca RC, Ong AA, Albasha O, et al. Photobiomodulation therapy for wound care: a potent, noninvasive, photoceutical approach. Adv Skin Wound Care. 2019;32(4):157–167. doi: 10.1097/01.ASW.0000553600.97572.d2. 30889017

[27] Karu TI, Afanas’eva NI. Cytochrome c oxidase as the primary photoacceptor upon laser exposure of cultured cells to visible and near IR-range light. Dokl Akad Nauk. 1995;342(5):693–695. 7670387

[28] Koceva I, Rümmelein B, Gerber PA, et al. Fluorescent light energy: a new therapeutic approach to effectively treating acne conglobata and hidradenitis suppurativa. Clin Case Rep. 2019;7(9):1769–1772. doi: 10.1002/ccr3.2334. 31534746 PMC6745390

[29] Sannino M, Lodi G, Dethlefsen MW, et al. Fluorescent light energy: treating rosacea subtypes 1, 2, and 3. Clin Case Rep. 2018;6(12):2385–2390. doi: 10.1002/ccr3.1891. 30564333 PMC6293188

[30] Scapagnini G, Marchegiani A, Rossi G, et al. Management of all three phases of wound healing through the induction of fluorescence biomodulation using fluorescence light energy. Proc. SPIE 10863, photonic diagnosis and treatment of infections and ­inflammatory diseases II, 108630W, San Francisco, California, United States, 7 March 2019, 2019. Bellingham (WA): International Society for Optics and Photonics.

[31] Mellergaard M, Fauverghe S, Scarpa C, et al. Evaluation of fluorescent light energy for the treatment of acute second-degree burns. Mil Med. 2021;186(Suppl 1):416–423. doi: 10.1093/milmed/usaa299. 33499452

[32] Guerra A, Belinha J, Jorge RN. Modelling skin wound healing angiogenesis: a review. J Theor Biol. 2018;459:1–17. doi: 10.1016/j.jtbi.2018.09.020. 30240579

[33] Ding JIE, Mellergaard M, Zhu Z, et al. Fluorescent light energy modulates healing in skin grafted mouse model. Open Med (Wars). 2021;16(1):1240–1255. doi: 10.1515/med-2021-0329. 34522783 PMC8402934

